# Inter-reader agreement in multimodality imaging of carotid plaque-RADS: considerations for modality selection

**DOI:** 10.1186/s13244-026-02393-3

**Published:** 2026-09-02

**Authors:** Jie Zhang, Yuzhu Yan, Yingqi Xing, Liyang Bai, Rina Sa, Lijuan Wang

**Affiliations:** 1 Neuroscience Center, Department of Neurology, First Hospital of Jilin University, Jilin University, Changchun, China; 2 Department of Radiology, First Hospital of Jilin University, Jilin University, Changchun, China; 3 Department of Vascular Ultrasonography, Xuanwu Hospital, Capital Medical University, Beijing, China

**Keywords:** Plaque-RADS, Inter-reader agreement, Ultrasound, Computed tomography angiography, Magnetic resonance imaging

## Abstract

**Objectives:**

Plaque-RADS was recently developed to standardize reporting and risk stratification of carotid plaques. This study aimed to evaluate inter-reader agreement of Plaque-RADS across US, CTA, and MRI, and to assess the impact of reader experience.

**Materials and methods:**

In 250 patients with carotid US, CTA, and MRI, nine readers (three per modality, stratified by experience) independently assessed plaque features and Plaque-RADS scores. Inter-reader agreement was analyzed using κ statistics and intraclass correlation coefficients (ICC). Learning curves were generated by comparing reader performance against MRI expert reference across five sequential case subsets.

**Results:**

Among 962 plaques and 25 normal arteries, inter-modality agreement for overall Plaque-RADS was moderate to substantial (κ, 0.578–0.758). Agreement was almost perfect for RADS 1-2 (κ, 0.814–0.883) but lower for RADS 3-4 (κ, 0.427–0.709). US, CTA, and MRI demonstrated substantial to almost perfect agreement in assessing plaque presence and maximum wall thickness (κ/ICC, 0.697–0.878). CTA and MRI experts demonstrated substantial to almost perfect agreement in ulcer detection (κ, 0.880) and intraluminal thrombus (κ, 0.787), but moderate agreement in intraplaque hemorrhage (κ, 0.578). Regarding reader experience, all intermediate and expert readers across US, CTA, and MRI achieved substantial to almost perfect agreement in overall and different categories of RADS scoring (κ/ICC, 0.624–0.954). Learning curve analysis confirmed that all readers improved with training, with the most pronounced gains observed in US beginners.

**Conclusion:**

Plaque-RADS is reliable for low-risk plaques across modalities, whereas accurate categorization of high‑risk plaques requires experienced readers and advanced imaging. These findings may help inform stratified imaging decisions.

**Key Points:**

- **Question** Does inter‑reader agreement for the Carotid Plaque‑RADS score differ across ultrasound, CTA, and MRI, and how does reader experience affect consistency?
- **Findings** Plaque‑RADS showed excellent agreement for low‑risk plaques across all modalities. High‑risk plaques required advanced imaging and experienced readers; structured training improved performance, especially for ultrasound.
- **Critical Relevance Statement** This study systematically compares Plaque‑RADS agreement across ultrasound, CTA, and MRI. The findings support a stratified imaging strategy (US for low‑risk assessment, MRI for high‑risk plaque characterization) pending prospective validation.

**Graphical Abstract:**

**Figure:**
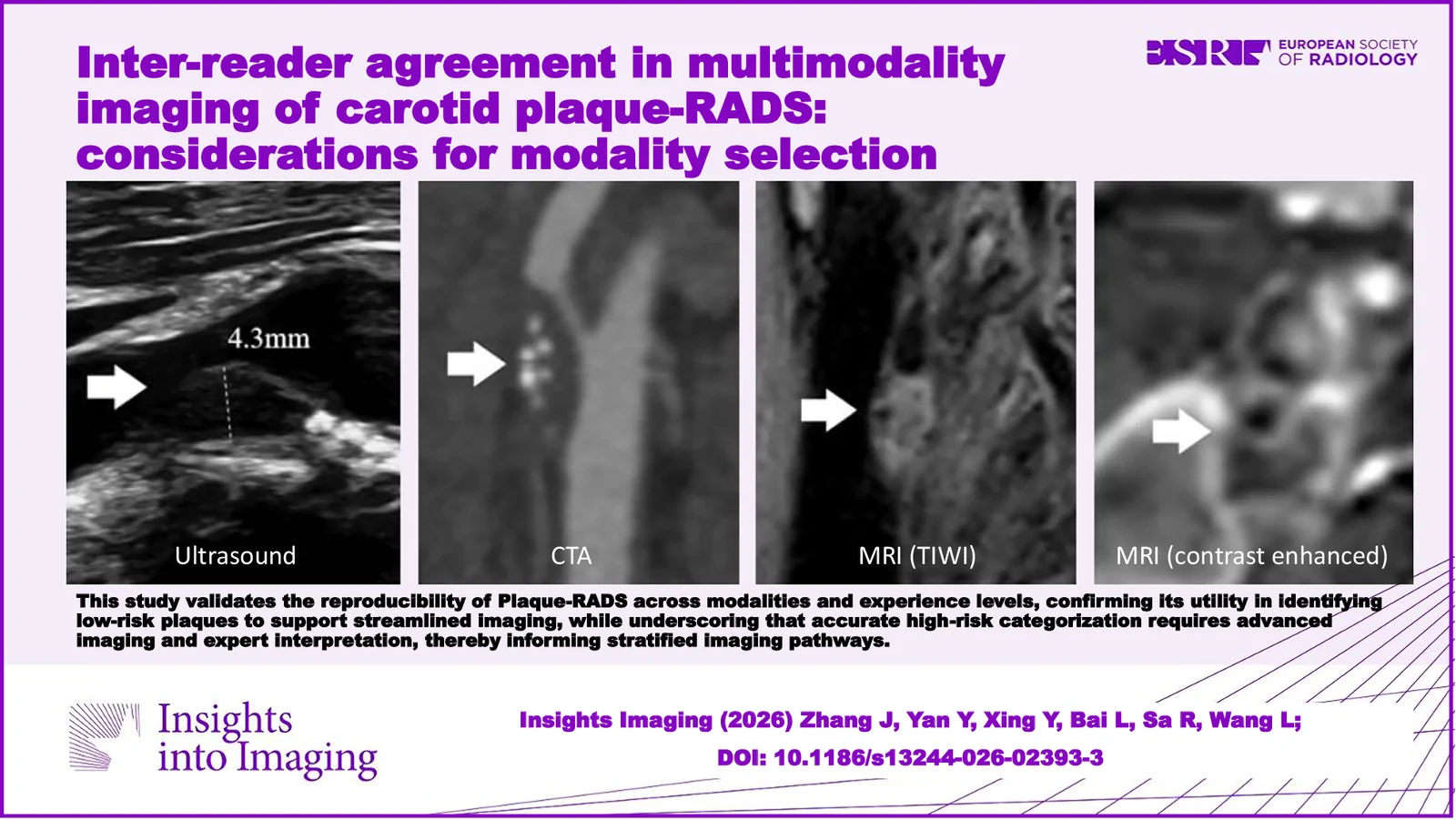

## Introduction

Carotid artery atherosclerosis is highly prevalent and a major cause of ischemic stroke. Traditional risk assessment has relied heavily on quantifying luminal stenosis. However, accumulating evidence indicates that plaque morphology and composition, rather than stenosis alone, are more critical determinants of its vulnerability and subsequent stroke risk [1]. Consequently, several imaging biomarkers have been developed to characterize plaque morphology and composition [2]. In 2024, the Carotid Plaque Reporting and Data System (Plaque-RADS) was introduced to standardize reporting and improve risk stratification. This cross‑modality classification system categorizes plaques from RADS 1 (no atherosclerosis) to RADS 4 (complicated plaque with high‑risk features) across multiple imaging modalities [3]. Validation studies have demonstrated that higher Plaque-RADS categories are strongly associated with increased risk of future ischemic events, offering significant net reclassification improvement over stenosis measurement alone [4].

Multiple imaging modalities are routinely used for carotid plaque characterization. Ultrasound (US) is widely accessible and cost-effective, rendering it the preferred initial screening tool [5]. Computed tomography angiography (CTA) provides high spatial resolution, excelling in the depiction of calcification and stenosis. Magnetic resonance imaging (MRI), especially high-resolution vessel wall imaging, is regarded as the non-invasive reference standard for plaque composition analysis due to its superior soft-tissue contrast, which enables excellent differentiation of intraplaque hemorrhage (IPH), lipid-rich necrotic core (LRNC), and fibrous cap integrity [6]. Each modality has distinct advantages and limitations; hence, a multimodal approach is often employed for comprehensive plaque evaluation [7]. Plaque-RADS provides a standardized system for reporting findings across these imaging techniques and is intended for use by sonographers, CT and MR specialists, and other radiologists to ensure consistent assessment. However, the reproducibility of image interpretation across different modalities remains incompletely understood. Although previous studies have reported favorable agreement in stenosis measurements between US and CTA, the consistency in classifying Plaque-RADS categories and high-risk plaque features warrants further investigation [8, 9]. Moreover, the influence of reader experience on the reliability of Plaque-RADS across US, CTA, and MRI has not been sufficiently examined. Similarly, the learning curve for achieving proficiency in interpreting these complex images remains undefined. Addressing these questions is critical to guiding optimal modality selection and resource allocation in carotid artery disease.

Therefore, this study aimed to evaluate and compare inter-reader agreement for Carotid Plaque-RADS across US, CTA, and MRI, and to assess how reader experience affects scoring consistency, while employing learning curve analysis to determine the number of cases required for less experienced readers to achieve diagnostic proficiency.

## Materials and methods

### Study population

**Fig. 1:**
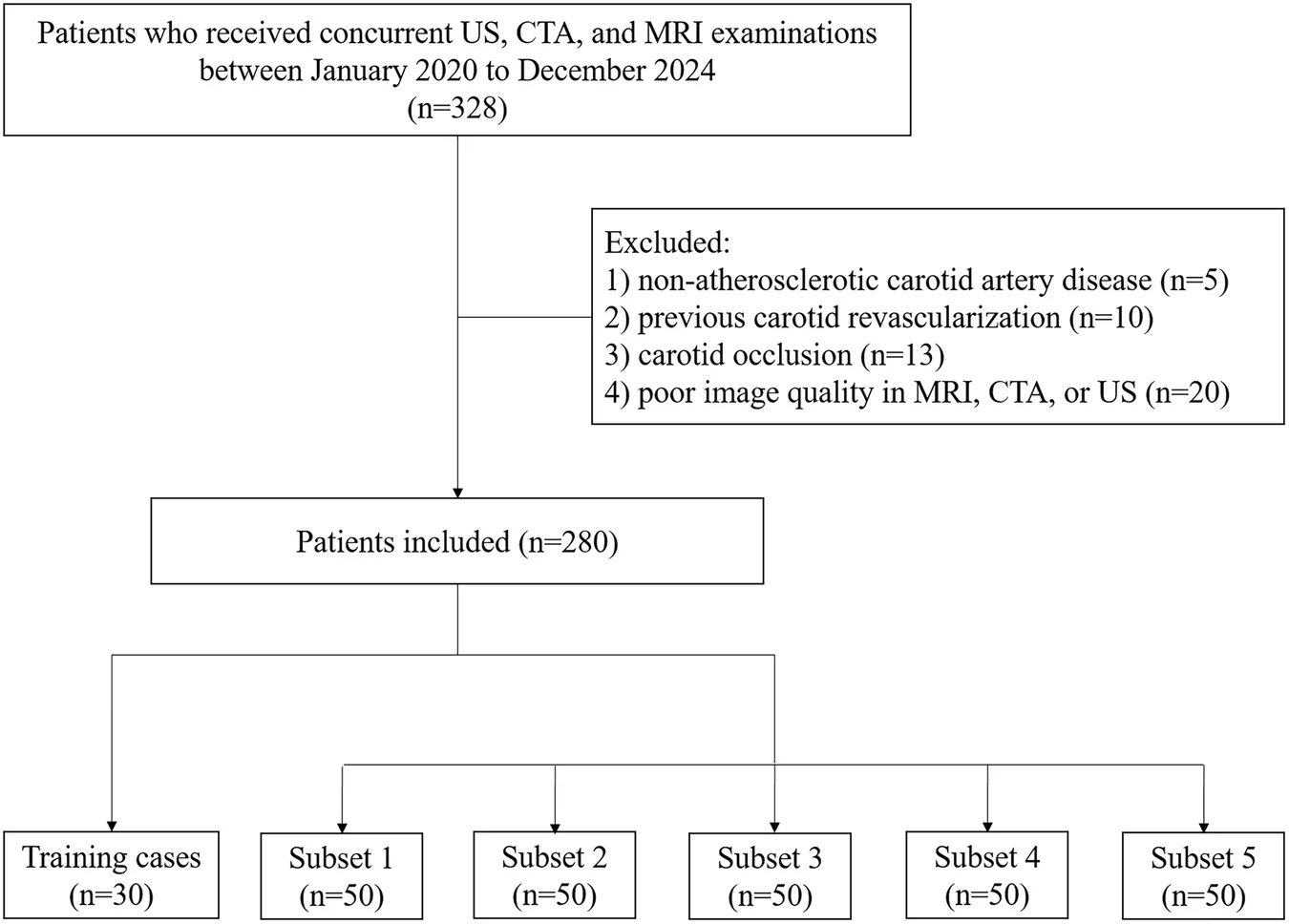
Study flowchart

The study protocol was approved by the Institutional Review Board of First Hospital of Jilin University (Approval Number: 2025-474) on September 23, 2025, which waived the requirement for written informed consent. From January 2020 to December 2024, all patients who received US, CTA, and MRI examinations within one month at the hospital were retrospectively enrolled. Multimodal imaging was clinically indicated to resolve diagnostic uncertainty and to formulate a definitive management plan. Exclusion criteria were as follows: (1) non-atherosclerotic carotid artery disease; (2) previous carotid revascularization; (3) carotid occlusion; and (4) poor image quality in MRI, CTA, or US. Clinical information was extracted from electronic medical records at the time of imaging examination. A detailed flowchart is shown in Fig. 1.

### Carotid image interpretation

All patients underwent standardized carotid artery imaging with US, CTA, and MRI. Detailed protocols are provided in Supplementary File S1. Images were anonymized and archived in the Picture Archiving and Communication System (PACS). Image interpretation and Plaque‑RADS scoring were performed between October and November 2025, after the Plaque‑RADS system had been published and after readers had completed standardized training. Nine readers (three per modality), stratified by experience (beginner < 6 months, intermediate 3–5 years, expert ≥ 10 years), independently evaluated the images. These cut‑offs were chosen to ensure clear separation between groups and to avoid overlap. The readers were blinded to patients’ clinical data and subsequent outcomes, to the prior imaging reports, and to the scores assigned by other readers. Readers first assessed major plaque features, then independently assigned Plaque‑RADS scores. For each lesion, they documented: (1) plaque presence; (2) maximum wall thickness (MWT); (3) ulceration; (4) fibrous cap integrity; (5) IPH; (6) intraluminal thrombus; and (7) final Plaque‑RADS score. Fibrous cap integrity was assessed only by US and MRI, as CTA cannot visualize the fibrous cap and US cannot reliably measure its thickness. Diagnostic criteria for each feature and modality are provided in Supplementary Table S1. Because US and CTA cannot reliably evaluate certain RADS subtypes, plaques were classified into the four main categories: Plaque-RADS 1: no plaque present; Plaque-RADS 2: plaque present with MWT < 3 mm and absence of ulceration or complicated features (fibrous cap rupture, IPH, intraluminal thrombus); Plaque-RADS 3: plaque with either MWT ≥ 3 mm or presence of ulceration, in the absence of complicated features; Plaque-RADS 4: presence of complicated features, irrespective of MWT. Only plaques clearly visible on all three modalities were included for feature and score recording.

### Training interventions

Before the formal Plaque-RADS assessment, a structured training program was conducted under the guidance of expert readers, with participation from all intermediate and beginner readers. The program began with a detailed explanation of the definitions for major plaque features. After confirming that all readers understood the above features, the specific criteria and classification rules for the Plaque-RADS scoring system were introduced and further elaborated using 30 representative cases with multimodal imaging. All cases used in training were excluded from the subsequent formal analysis.

The enrolled cases were randomly divided into five subsets. No significant difference was observed in the distribution of Plaque‑RADS categories across the five subsets (Supplemental Table S2). Each trainee independently interpreted the images in each subset. After completion of a subset, the trainee received feedback from the trainer, engaged in thorough discussion, and then proceeded to the next subset. The reference standard was derived from a consensus between two MRI experts, one participating reader from the main study and an independent expert with over 20 years of carotid artery imaging experience. In cases of disagreement, consensus was reached through joint review and discussion. The inter-reader agreement between these two MRI experts before consensus was almost perfect, with a Cohen’s kappa coefficient (κ) of 0.950. All reading and training sessions were completed within two months.

### Statistical analysis

Sample size was calculated using the confidence interval (CI) approach for a four-category outcome. Assuming an expected κ of 0.4 for the four‑category Plaque‑RADS score, with category proportions of 0.05, 0.15, 0.40 and 0.40 derived from a pilot study, and desiring a 95% CI width of 0.10 (α = 0.05), a total of 836 plaques was required. Continuous data are presented as mean ± standard deviation (SD), and categorical data as frequencies (percentages). Inter‑reader agreement for binary or nominal variables was assessed using unweighted Cohen’s κ with 95%CI. For ordinal Plaque‑RADS categories, quadratically weighted κ was used. The intraclass correlation coefficient (ICC) with 95%CI was applied for continuous measurements. For feature‑modality pairs not reliably assessable due to inherent technical limitations, no agreement statistic was calculated (marked “NA” in tables). For each imaging modality, three readers (beginner, intermediate, expert) were included. Rather than a single multi‑reader κ, we performed pairwise comparisons for specific reader and modality pairs, with 95%CIs obtained using the standard asymptotic method. Agreement was interpreted as: 0.81–1.00 (almost perfect), 0.61–0.80 (substantial), 0.41–0.60 (moderate), 0.21–0.40 (fair), and ≤ 0.20 (slight). Learning curves were generated from κ statistics between each reader and the MRI expert reference across five sequential subsets. Reclassification percentages based on confusion matrices were calculated to evaluate classification gain when transitioning between modalities. To account for potential non‑independence due to multiple plaques within the same carotid artery, we performed a sensitivity analysis by randomly selecting one plaque per artery from the original dataset. This random selection was repeated 100 times (using a fixed random seed for reproducibility). For each iteration, all main analyses, including inter‑reader agreement for major features, Plaque‑RADS categories, and learning curves, were recalculated using the reduced dataset (one plaque per artery). The agreement values were then compared with the primary analysis based on all plaques. To separate modality‑intrinsic limitations from reader experience, we additionally benchmarked each reader against the expert reader of the same imaging modality and reconstructed the learning curves using the same sequential subset approach. Statistical analyses were performed using SPSS (version 27.0; IBM) and R (version 4.2.0; The R Foundation for Statistical Computing).

## Results

### Patients’ characteristics

A total of 250 patients were enrolled (176 men, 74 women; mean age 65.9 ± 7.3 years). Comorbidities included hypertension (152 patients, 60.8%), diabetes mellitus (69, 27.6%), dyslipidemia (86, 34.4%), stroke history (195, 78.0%), coronary artery disease (100, 40.0%), and smoking (192, 76.8%). The analysis encompassed 500 carotid arteries, of which 25 were classified as normal, and 475 contained atherosclerotic plaques, yielding a total of 962 plaques for evaluation. Using expert MRI readings as the reference, the distribution was as follows: among 500 carotid arteries, 25 (5.0%) were classified as Plaque‑RADS 1. Among the 962 plaques identified, 265 (27.5%) were RADS 2, 376 (39.1%) were RADS 3, and 321 (33.4%) were RADS 4.

### Inter-reader agreement on major features

**Fig. 2:**
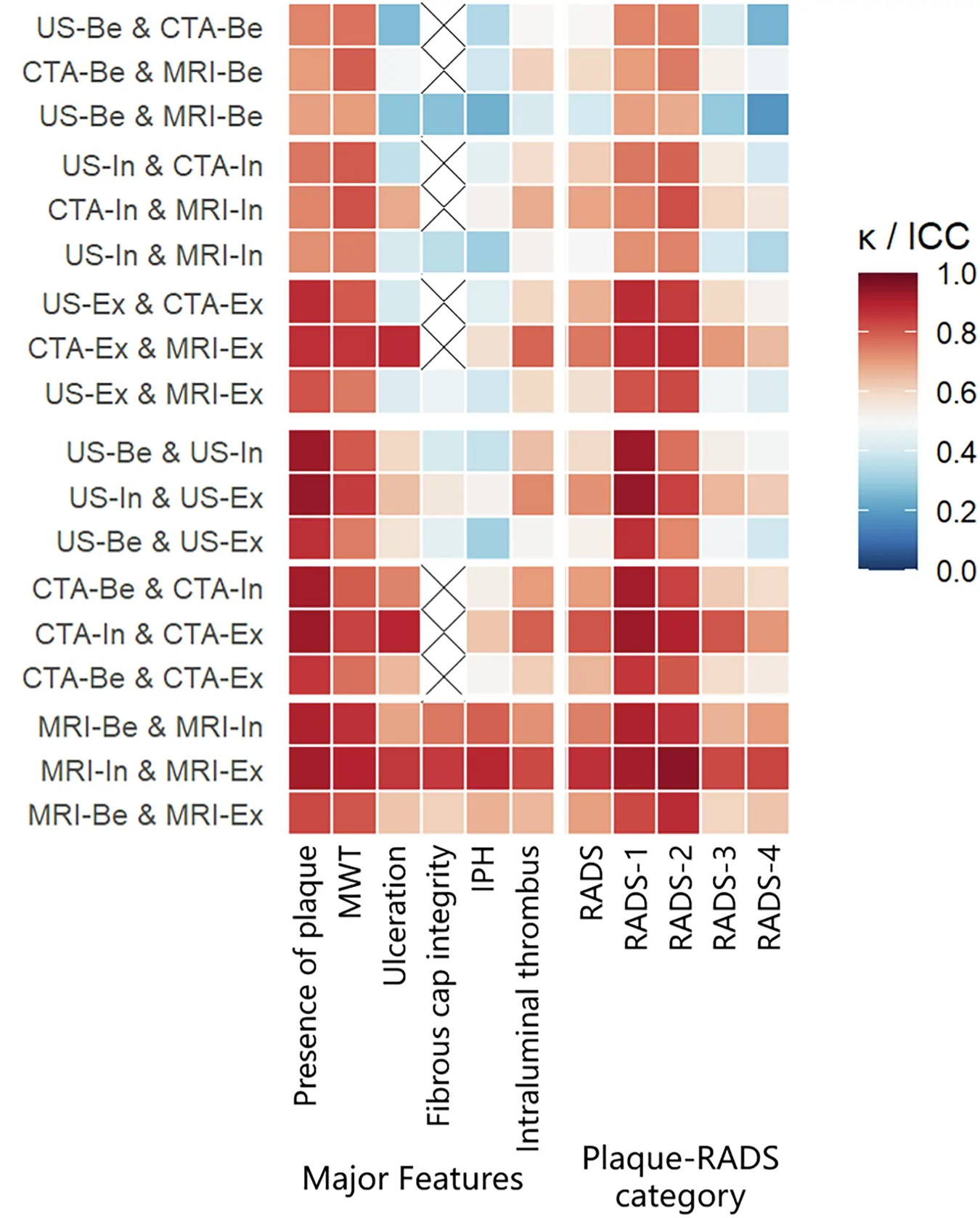
Heatmap of inter‑reader agreement for Plaque‑RADS scoring and major plaque features across imaging modalities and reader experience levels. Colors represent κ or ICC values: red indicates higher agreement, blue indicates lower agreement. A cross (×) denotes that the imaging modality cannot reliably assess the feature. Be, beginner readers; In, intermediate readers; Ex, expert readers; US, ultrasound; CTA, computed tomography angiography; MRI, magnetic resonance imaging; MWT, maximum wall thickness; IPH, intraplaque hemorrhage; Plaque-RADS, carotid plaque reporting and data system

Agreement for major features across modalities and experience levels is shown in Fig. 2 and Table 1. Across modalities, US, CTA, and MRI showed substantial to almost‑perfect agreement for plaque presence (κ, 0.697–0.878) and MWT (ICC, 0.703–0.863). For ulceration, US vs CTA/MRI experts showed moderate agreement (κ, 0.412–0.426), while CTA vs MRI experts showed almost‑perfect agreement (κ, 0.880). Fibrous cap integrity was moderate between US and MRI experts (κ, 0.469). For IPH, agreement was moderate for US vs CTA (κ, 0.438) and CTA vs MRI (κ, 0.578), and fair for US vs MRI (κ, 0.397). For intraluminal thrombus, agreement was substantial for US vs CTA (κ, 0.602) and CTA vs MRI (κ, 0.787), and moderate for US vs MRI (κ, 0.596).

**Table 1:** Inter-reader agreement of the major features of Plaque-RADS

	Presence of plaque	MWT	Ulceration	Fibrous cap integrity	IPH	Intraluminal thrombus
Based on different imaging modalities						
Be						
US & CTA	0.737 (0.621, 0.853)	0.766 (0.738, 0.790)	0.269 (0.185, 0.353)	NA	0.346 (0.279, 0.413)	0.505 (0.340, 0.670)
CTA & MRI	0.705 (0.578, 0.832)	0.796 (0.769, 0.820)	0.483 (0.396, 0.569)	NA	0.395 (0.334, 0.456)	0.612 (0.437, 0.786)
US & MRI	0.697 (0.572, 0.822)	0.703 (0.663, 0.738)	0.288 (0.196, 0.380)	0.279 (0.199, 0.359)	0.242 (0.171, 0.313)	0.416 (0.244, 0.588)
In						
US & CTA	0.760 (0.652, 0.868)	0.804 (0.781, 0.825)	0.371 (0.271, 0.471)	NA	0.450 (0.372, 0.528)	0.585 (0.430, 0.740)
CTA & MRI	0.737 (0.617, 0.857)	0.816 (0.791, 0.837)	0.683 (0.606, 0.759)	NA	0.519 (0.446, 0.592)	0.675 (0.516, 0.834)
US & MRI	0.723 (0.603, 0.843)	0.747 (0.713, 0.777)	0.416 (0.310, 0.522)	0.361 (0.273, 0.449)	0.311 (0.237, 0.385)	0.522 (0.361. 0.683)
Ex						
US & CTA	0.878 (0.794, 0.962)	0.805 (0.782, 0.826)	0.412 (0.308, 0.516)	NA	0.438 (0.362, 0.514)	0.602 (0.412, 0.765)
CTA & MRI	0.874 (0.782, 0.966)	0.863 (0.845, 0.879)	0.880 (0.831, 0.929)	NA	0.578 (0.639, 0.517)	0.787 (0.669, 0.905)
US & MRI	0.814 (0.706, 0.922)	0.756 (0.724, 0.786)	0.426 (0.320, 0.532)	0.469 (0.382, 0.555)	0.397 (0.319, 0.475)	0.596 (0.441. 0.751)
Based on different reader experiences						
US						
Be & In	0.932 (0.873, 0.991)	0.806 (0.785, 0.825)	0.599 (0.430, 0.768)	0.412 (0.304, 0.520)	0.378 (0.307, 0.449)	0.644 (0.491, 0.797)
In & Ex	0.940 (0.887, 0.993)	0.850 (0.832, 0.867)	0.645 (0.496, 0.794)	0.558 (0.476, 0.640)	0.519 (0.446, 0.592)	0.733 (0.587, 0.878)
Be & Ex	0.873 (0.795, 0.951)	0.751 (0.723, 0.777)	0.570 (0.411, 0.729)	0.449 (0.367, 0.531)	0.316 (0.245, 0.386)	0.511 (0.333, 0.689)
CTA						
Be & In	0.925 (0.860, 0.990)	0.800 (0.776, 0.821)	0.740 (0.671, 0.809)	NA	0.532 (0.465, 0.599)	0.705 (0.509, 0.901)
In & Ex	0.932 (0.875, 0.989)	0.838 (0.818, 0.855)	0.893 (0.846, 0.940)	NA	0.633 (0.568, 0.698)	0.793 (0.617, 0.969)
Be & Ex	0.860 (0.774, 0.946)	0.770 (0.734, 0.803)	0.659 (0.583, 0.735)	NA	0.509 (0.444, 0.574)	0.620 (0.438, 0.802)
MRI						
Be & In	0.907 (0.825, 0.989)	0.872 (0.856, 0.886)	0.690 (0.614, 0.766)	0.759 (0.702, 0.816)	0.792 (0.729, 0.855)	0.720 (0.567, 0.872)
In & Ex	0.920 (0.851, 0.989)	0.899 (0.886, 0.910)	0.855 (0.800, 0.910)	0.853 (0.804, 0.902)	0.888 (0.843, 0.933)	0.829 (0.731, 0.927)
Be & Ex	0.828 (0.724, 0.932)	0.810 (0.788, 0.831)	0.635 (0.555, 0.715)	0.614 (0.543, 0.685)	0.669 (0.593, 0.745)	0.658 (0.505, 0.811)

Upper section (“Based on different imaging modalities”): agreement estimates are derived from different readers for each modality. Therefore, these values reflect combined inter‑modality and inter‑reader variability and cannot separate technical differences between modalities from reader‑dependent disagreement

Lower section (“Based on different reader experiences”): agreement estimates are derived from readers of the same modality. These values represent pure inter‑reader agreement within each imaging technique

Given that CTA could not directly evaluate fibrous cap integrity, this parameter was not assessed in the CTA analysis

Data are kappa scores or ICC. Data in parentheses are 95% CIs

*MWT* maximal wall thickness, *IPH* intra-plaque hemorrhage, *Be* beginner readers, *In* intermediate readers, *Ex* expert readers, *US* ultrasound, *CTA* computed tomography angiography, *MRI* magnetic resonance imaging, *NA* not applicable

Across experience levels, all groups showed substantial to almost‑perfect agreement for plaque presence (κ, 0.828–0.940) and MWT (ICC, 0.751–0.899). Ulceration agreement was moderate‑to‑substantial for US (κ, 0.570–0.645) vs substantial‑to‑almost‑perfect for CTA (κ, 0.659–0.893) and MRI (κ, 0.635–0.855). Fibrous cap integrity was moderate among US readers (κ, 0.412–0.558) but substantial‑to‑almost‑perfect among MRI readers (κ, 0.614–0.853). IPH agreement ranged from fair‑to‑moderate (US, κ, 0.316–0.519) to moderate‑to‑substantial (CTA, κ, 0.509–0.633) and substantial‑to‑almost‑perfect (MRI, κ, 0.669–0.888). Intraluminal thrombus agreement ranged from moderate‑to‑substantial (US, κ, 0.511–0.733) to substantial (CTA, κ, 0.620–0.793) and substantial‑to‑almost‑perfect (MRI, κ, 0.658–0.829).

### Inter-reader agreement on plaque-RADS categories

Agreement for Plaque‑RADS scoring is shown in Fig. 2 and Table 2. Across modalities, US vs CTA experts showed substantial agreement (κ, 0.669), CTA vs MRI experts showed substantial agreement (κ, 0.758), and US vs MRI experts showed moderate agreement (κ, 0.578). Experts across all modalities showed almost‑perfect agreement for RADS‑1‑2 (κ, 0.814–0.883). For RADS‑3‑4, CTA vs MRI maintained substantial agreement (κ, 0.653–0.709).

**Table 2:** Inter-reader agreement for assigning plaque-RADS categories

	RADS	RADS-1	RADS-2	RADS-3	RADS-4
Based on different imaging modalities					
Be					
US and CTA	0.506 (0.463, 0.549)	0.737 (0.621, 0.853)	0.749 (0.704, 0.794)	0.414 (0.371, 0.457)	0.253 (0.506, 0.654)
CTA & MRI	0.595 (0.554, 0.636)	0.705 (0.578, 0.832)	0.753 (0.708, 0.798)	0.527 (0.472, 0.582)	0.475 (0.414, 0.536)
US & MRI	0.404 (0.361, 0.447)	0.697 (0.572, 0.822)	0.679 (0.630, 0.728)	0.296 (0.239, 0.353)	0.185 (0.124, 0.246)
In					
US & CTA	0.620 (0.579, 0.661)	0.760 (0.652, 0.868)	0.788 (0.745, 0.831)	0.550 (0.499, 0.601)	0.409 (0.340, 0.478)
CTA & MRI	0.691 (0.652, 0.730)	0.737 (0.617, 0.857)	0.821 (0.782, 0.860)	0.607 (0.554, 0.660)	0.561 (0.504, 0.618)
US & MRI	0.502 (0.460, 0.546)	0.723 (0.603, 0.843)	0.742 (0.695, 0.789)	0.405 (0.350, 0.460)	0.341 (0.278, 0.404)
Ex					
US & CTA	0.669 (0.630, 0.708)	0.878 (0.794, 0.962)	0.851 (0.814, 0.888)	0.596 (0.547, 0.645)	0.521 (0.458, 0.584)
CTA & MRI	0.758 (0.727, 0.789)	0.874 (0.782, 0.966)	0.883 (0.850, 0.916)	0.709 (0.666, 0.752)	0.653 (0.608, 0.698)
US & MRI	0.578 (0.537, 0.619)	0.814 (0.706, 0.922)	0.829 (0.790, 0.868)	0.480 (0.427, 0.533)	0.427 (0.368, 0.486)
Based on different reader experiences					
US					
Be & In	0.594 (0.555, 0.633)	0.932 (0.873, 0.991)	0.768 (0.725, 0.811)	0.533 (0.484, 0.582)	0.486 (0.408, 0.564)
In & Ex	0.720 (0.687, 0.753)	0.940 (0.887, 0.993)	0.842 (0.812, 0.872)	0.659 (0.618, 0.700)	0.624 (0.557, 0.691)
Be & Ex	0.526 (0.485, 0.567)	0.873 (0.795, 0.951)	0.735 (0.690, 0.780)	0.482 (0.431, 0.433)	0.395 (0.317, 0.473)
CTA					
Be & In	0.704 (0.665, 0.743)	0.925 (0.860, 0.990)	0.841 (0.804, 0.878)	0.625 (0.576, 0.674)	0.591 (0.526, 0.656)
In & Ex	0.812 (0.783, 0.841)	0.932 (0.875, 0.989)	0.903 (0.874, 0.932)	0.812 (0.775, 0.849)	0.711 (0.668, 0.754)
Be & Ex	0.662 (0.623, 0.701)	0.860 (0.774, 0.946)	0.807 (0.766, 0.848)	0.589 (0.538, 0.640)	0.546 (0.485, 0.607)
MRI					
Be & In	0.746 (0.706, 0.780)	0.907 (0.825, 0.989)	0.870 (0.835, 0.905)	0.666 (0.620, 0.722)	0.702 (0.644, 0.750)
In & Ex	0.870 (0.845, 0.895)	0.920 (0.851, 0.989)	0.954 (0.932, 0.976)	0.829 (0.794, 0.864)	0.836 (0.802, 0.876)
Be & Ex	0.699 (0.662, 0.736)	0.828 (0.724, 0.932)	0.879 (0.846, 0.912)	0.607 (0.556, 0.658)	0.635 (0.582, 0.688)

Upper section (“Based on different imaging modalities”): agreement estimates are derived from different readers for each modality. Therefore, these values reflect combined inter‑modality and inter‑reader variability and cannot separate technical differences between modalities from reader‑dependent disagreement

Lower section (“Based on different reader experiences”): agreement estimates are derived from readers of the same modality. These values represent pure inter‑reader agreement within each imaging technique

Data are kappa scores or ICC. Data in parentheses are 95% CIs

*Be* beginner readers, *In* intermediate readers, *Ex* expert readers, *US* ultrasound, *CTA* computed tomography angiography, *MRI* magnetic resonance imaging

**Fig. 3:**
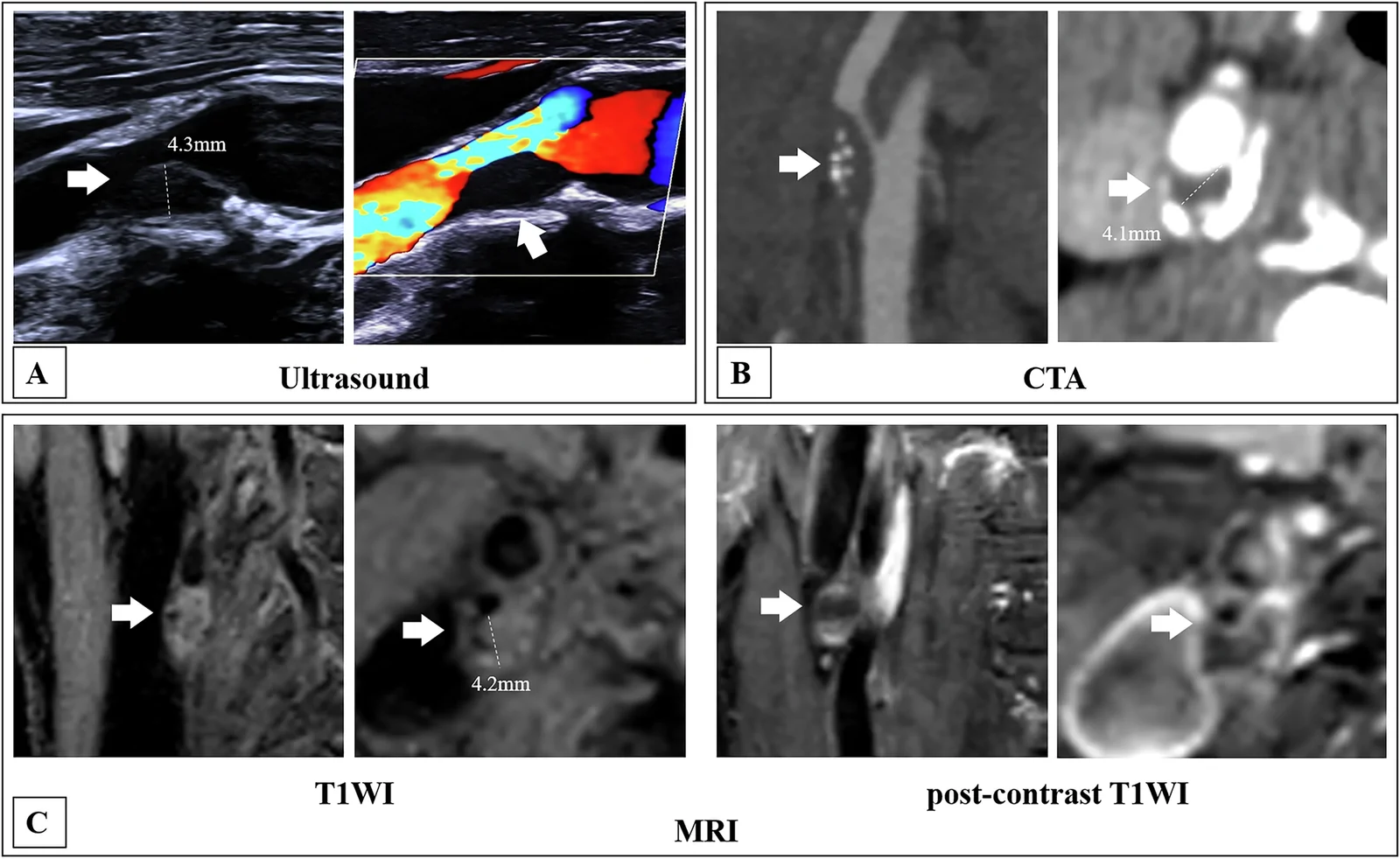
A 70-year-old male with severe stenosis of the right internal carotid artery, classified as Plaque-RADS score 4. **A** US reveals an eccentric plaque (arrow) in the proximal right internal carotid artery, demonstrating a maximal wall thickness of 4.3 mm, a gray-scale median of 20, and a juxtaluminal black area. **B** CTA identifies the plaque (arrow) as a mixed type, featuring a hypodense core with a measurement of 19 HU and maximal wall thickness of 4.1 mm. **C** MRI demonstrates that the plaque (arrow) has moderately high signal intensity on T1WI and shows no enhancement on post-contrast T1WI, findings which are consistent with IPH

Across experience levels, overall RADS agreement was moderate‑to‑substantial for US readers (κ, 0.526–0.720) and substantial‑to‑almost‑perfect for CTA (κ, 0.662–0.812) and MRI readers (κ, 0.699–0.870). RADS‑1‑2 agreement was substantial‑to‑almost‑perfect across all levels and modalities (κ, 0.735–0.954). For RADS‑3, almost‑perfect agreement was observed for CTA intermediate‑expert (κ, 0.812) and MRI intermediate‑expert (κ, 0.829). For RADS‑4, only MRI intermediate‑expert maintained almost‑perfect agreement (κ, 0.836). A representative RADS‑4 case is shown in Fig. 3.

### Learning curve

**Fig. 4:**
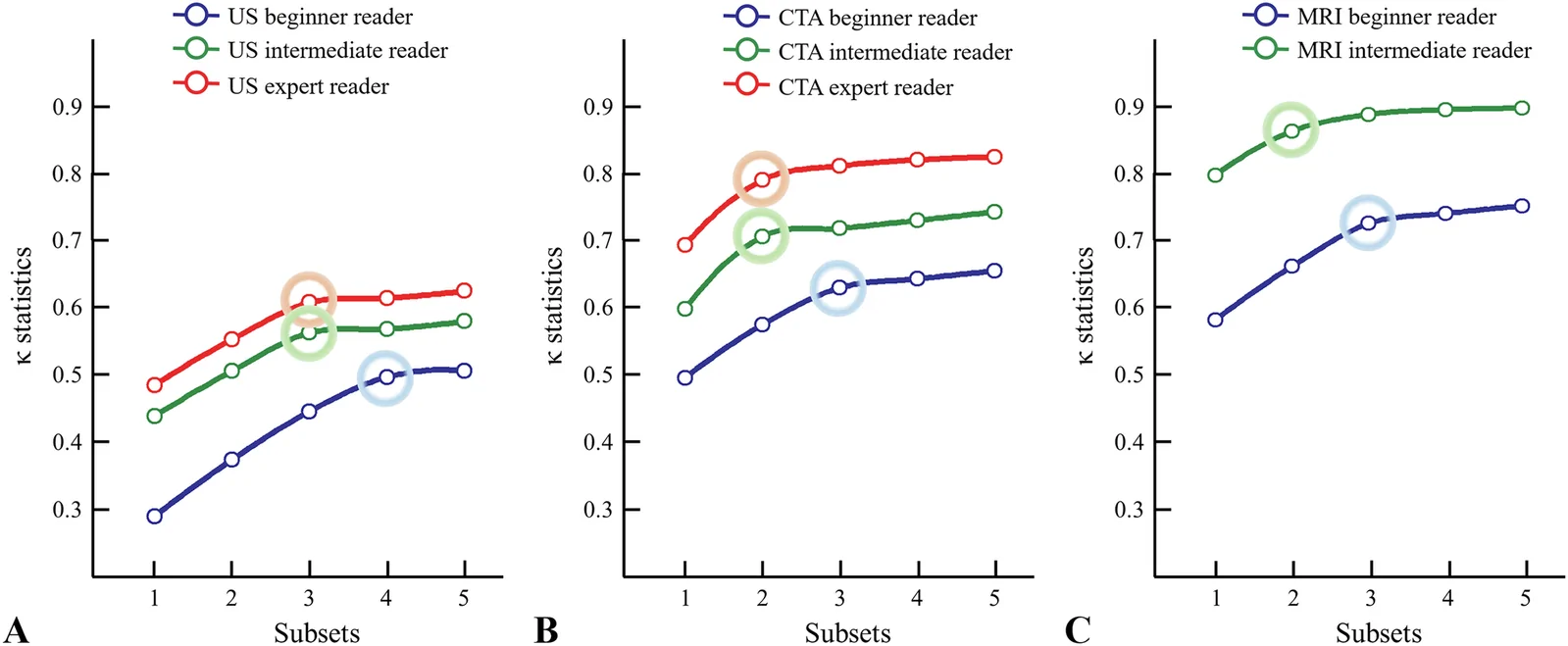
Learning curves for Plaque‑RADS scoring. Each curve represents the quadratically weighted κ for agreement between a given reader and the MRI expert reference, plotted across five sequential subsets (50 patients per subset; cumulative totals of 50, 100, 150, 200, and 250 patients). Plateau points are indicated by circles. **A** Ultrasound readers; **B** CTA readers; **C** MRI readers

Figure 4 and Table 3 shows the κ statistics between each reader and the MRI expert reference for each subset. The κ statistic between the US beginner reader and the reference standard improved steeply from 0.290 in subset 1 (first 50 patients) to 0.497 in subset 4 (cumulative 200 patients), followed by a slight improvement to 0.506 in subset 5 (cumulative 250 patients). A similar upward trend in agreement was observed across all reader groups over successive subsets. The learning curves plateaued at different stages: for US intermediate, US expert, CTA beginner, and MRI beginner readers, plateaus occurred in subset 3 (cumulative 150 patients); for CTA intermediate, CTA expert, and MRI intermediate readers, plateaus were reached by subset 2 (cumulative 100 patients). These findings suggest that for most readers, basic proficiency in Plaque‑RADS scoring can be achieved after interpreting approximately 100–150 cases. Notably, US beginners continued to improve across the first four subsets, indicating that around 200 cases may be required to reach a stable performance plateau, highlighting the greater operator dependence of US.

**Table 3:** Inter-reader agreement across sequential subsets

κ statistics	Subset 1	Subset 2	Subset 3	Subset 4	Subset 5
US beginner reader	0.290 (0.189, 0.392)	0.374 (0.280, 0.468)	0.446 (0.354, 0.538)	0.497 (0.403, 0591)	0.506 (0.414, 0.598)
US intermediate reader	0.439 (0.343, 0.535)	0.506 (0.410, 0.602)	0.563 (0.469, 0.657)	0.568 (0.478, 0.658)	0.580 (0.490, 0.670)
US expert reader	0.485 (0.389, 0.581)	0.553 (0.461, 0.645)	0.608 (0.518, 0.698)	0.614 (0.526, 0.702)	0.625 (0.539, 0.713)
CTA beginner reader	0.496 (0.400, 0.592)	0.575 (0.483, 0.667)	0.630 (0.540, 0.720)	0.643 (0.557, 0.729)	0.655 (0.569, 0.741)
CTA intermediate reader	0.598 (0.508, 0.688)	0.706 (0.624, 0.788)	0.718 (0.636, 0.800)	0.730 (0.652, 0.808)	0.743 (0.665, 0.821)
CTA expert reader	0.693 (0.609, 0.777)	0.790 (0.717, 0.863)	0.811 (0.770, 0.882)	0.820 (0.753, 0.887)	0.824 (0.757, 0.891)
MRI beginner reader	0.582 (0.486, 0.678)	0.662 (0.574, 0.750)	0.726 (0.646, 0.806)	0.741 (0.663, 0.819)	0.752 (0.676, 0.828)
MRI intermediate reader	0.798 (0.725, 0.871)	0.864 (0.803, 0.925)	0.889 (0.834, 0.944)	0.896 (0.843, 0.949)	0.898 (0.847, 0.949)

*US* ultrasound, *CTA* computed tomography angiography, *MRI* magnetic resonance imaging

### Reclassification analysis between imaging modalities

We assessed the gain in correct risk stratification when transitioning between US expert, CTA expert, and MRI expert. For RADS-1, all inter-modality reclassification percentages were minimal (US vs CTA: 0.9%; US vs MRI: 1.7%; CTA vs MRI: 1.2%), indicating no obvious improvement in classification accuracy across modalities. A similar pattern was observed for RADS-2 plaques, where reclassification percent remained low (US vs CTA: 6.2%; US vs MRI: 7.0%; CTA vs MRI: 4.7%). In contrast, a marked increase in reclassification percent was observed for higher-risk categories. For RADS-3 plaques, it rose substantially, particularly for US vs MRI (30.9%), with moderate improvements for US vs CTA (20.3%) and slight improvements for CTA vs MRI (12.6%). This trend was most pronounced for RADS-4 plaques, where the reclassification percent for US vs MRI reached 40.5%, indicating substantial reclassification improvement. Meaningful gains were also seen for US vs CTA (29.6%) and CTA vs MRI (22.3%) in this category.

### Sensitivity analysis

First, to address potential clustering of multiple plaques per artery, we repeated all main analyses using one randomly selected plaque per artery (100 iterations). All agreement estimates and learning curve trends remained nearly unchanged, indicating that clustering did not materially affect our conclusions (Supplemental Tables S3–5). Second, to separate modality‑intrinsic limitations from reader experience, we reconstructed learning curves using the same‑modality expert as the reference. When benchmarked against the US expert, the US beginner reader achieved a substantially higher plateau (κ = 0.753) than against the MRI expert (κ = 0.497), confirming that the original curve partly reflected a modality ceiling. Similar patterns were observed for CTA readers, while learning trends and plateau points remained consistent across references (Supplemental Tables S6).

### Subcategory agreement analysis

We evaluated inter‑reader agreement for Plaque‑RADS subcategories (3a–3c, 4a–4c) using plaques reliably characterizable by each modality (Supplemental Table S7). For MRI readers, agreement ranged from moderate to almost perfect: 3a (κ, 0.763–0.865), 3b (0.641–0.791), 3c (0.599–0.817), 4a (0.774–0.888), 4b (0.614–0.853), and 4c (0.658–0.829). For CTA, only 3c, 4a, and 4c were assessable, with substantial to almost perfect agreement vs MRI experts (κ, 0.843, 0.674, and 0.787, respectively); 3a, 3b, and 4b were not evaluable. For US, only 3c and 4a–4c were partially assessable, with consistently lower agreement vs MRI experts (κ, 0.414, 0.285, 0.469, and 0.596, respectively).

## Discussion

This study evaluated inter-reader agreement for the Carotid Plaque-RADS scoring system across US, CTA, and MRI, incorporating multiple reader experience levels and learning curve analysis. Key findings included: (1) the Carotid Plaque-RADS achieved excellent reliability for lower-risk plaques across all modalities; (2) MRI remains the preferred modality for high-risk plaque characterization; and (3) although reader experience significantly influences agreement, particularly in US, structured training with defined case volumes effectively reduces variability. These results may help inform imaging selection in carotid artery disease, but prospective validation is needed before any pathway can be recommended.

While the conventional roles of US, CTA, and MRI in carotid imaging are established, comparative evidence under a standardized classification framework has remained limited. The recent introduction of the Carotid Plaque-RADS represents a significant advancement towards structured reporting. However, clear, evidence-based guidance on how to optimally select among these imaging modalities for specific Plaque-RADS grades remains undefined. By systematically evaluating inter-modality and inter-reader agreement, our study sought to identify scenarios in which each technique may perform more consistently, thereby offering reference to support the development of refined, evidence-informed imaging pathways.

Our findings indicate that differential modality performance across RADS categories supports a more tailored imaging strategy. The Carotid Plaque-RADS showed excellent agreement for RADS 1–2 plaques across all modalities (κ, 0.814–0.883). Negligible reclassification percentages confirm that once US identifies a low-risk plaque, CTA or MRI provides minimal incremental classification benefit. These observations support US as a first-line tool in resource-conscious settings, aligning with guideline recommendations (Class I, Level A) for initial assessment of suspected carotid stenosis [5]. The cost-effectiveness and wide availability of US further endorse its role in the initial evaluation and follow-up of low-risk patients in secondary prevention settings [10]. Moreover, consistently high agreement across experience levels suggests that with adequate training, less experienced readers can reliably interpret straightforward US cases, facilitating broader implementation of US as a first-line assessment tool in patients with suspected carotid disease.

For RADS 3 plaques, characterized by moderate risk features, our data reveal a more complex landscape. While CTA and MRI maintained substantial agreement (κ, 0.653–0.709), US agreement was lower (κ, 0.427–0.480). This suggests that after US identifies a moderate-risk plaque, confirmatory imaging with CTA or MRI should be considered, especially when management depends on precise characterization. The superior performance of CTA in ulcer detection (κ, 0.880 between CTA and MRI experts) makes it particularly valuable for RADS 3c plaques, where ulceration presence may influence treatment intensity [11, 12]. Its superiority in evaluating luminal stenosis and plaque surface characteristics makes it particularly valuable for procedural planning [13]. The substantial agreement between CTA and MRI experts for overall RADS scoring (κ, 0.758) also supports its role as a reliable alternative when MRI is not feasible.

In our study, the most significant modality-dependent variation emerged in RADS 4 plaque assessment, where MRI consistently outperformed other modalities (κ, 0.836 between MRI intermediate and expert readers). The steeply rising reclassification percentages for RADS-4 plaques, particularly for the US-to-MRI transition, quantify MRI’s definitive value in characterizing the highest-risk plaques. This stems from the inherent limitations of US and CTA: US cannot reliably assess fibrous cap integrity or IPH [14], while CTA cannot directly visualize the fibrous cap and shows only moderate agreement for IPH (κ, 0.578). Therefore, MRI is the preferred modality for definitive high-risk plaque characterization, especially when revascularization is considered [15, 16], as its superior soft-tissue contrast directly visualizes the features defining RADS 4 [1, 17, 18].

The profound impact of reader experience on interpretation consistency, particularly for US, represents a critical finding with direct implications for training and quality assurance. The dramatic improvement in agreement from beginner-expert (κ, 0.526) to intermediate-expert (κ, 0.720) for US underscores its operator-dependent nature. This experience effect was considerably less pronounced for CTA and MRI, where intermediate readers achieved near-expert performance levels. These differences likely reflect the more objective, less operator-dependent nature of cross-sectional imaging interpretation compared to the inherent subjectivity of US. The learning curve analysis provides tangible targets for training program development. The plateau points after 2–4 subsets (approximately 100–200 cases) offer the possible case requirements for achieving basic competency. The steeper learning curves for US beginners, while initially challenging, demonstrate that structured training can substantially improve performance. These findings strongly support the implementation of standardized training curricula with defined case volumes before independent Plaque-RADS interpretation.

**Fig. 5:**
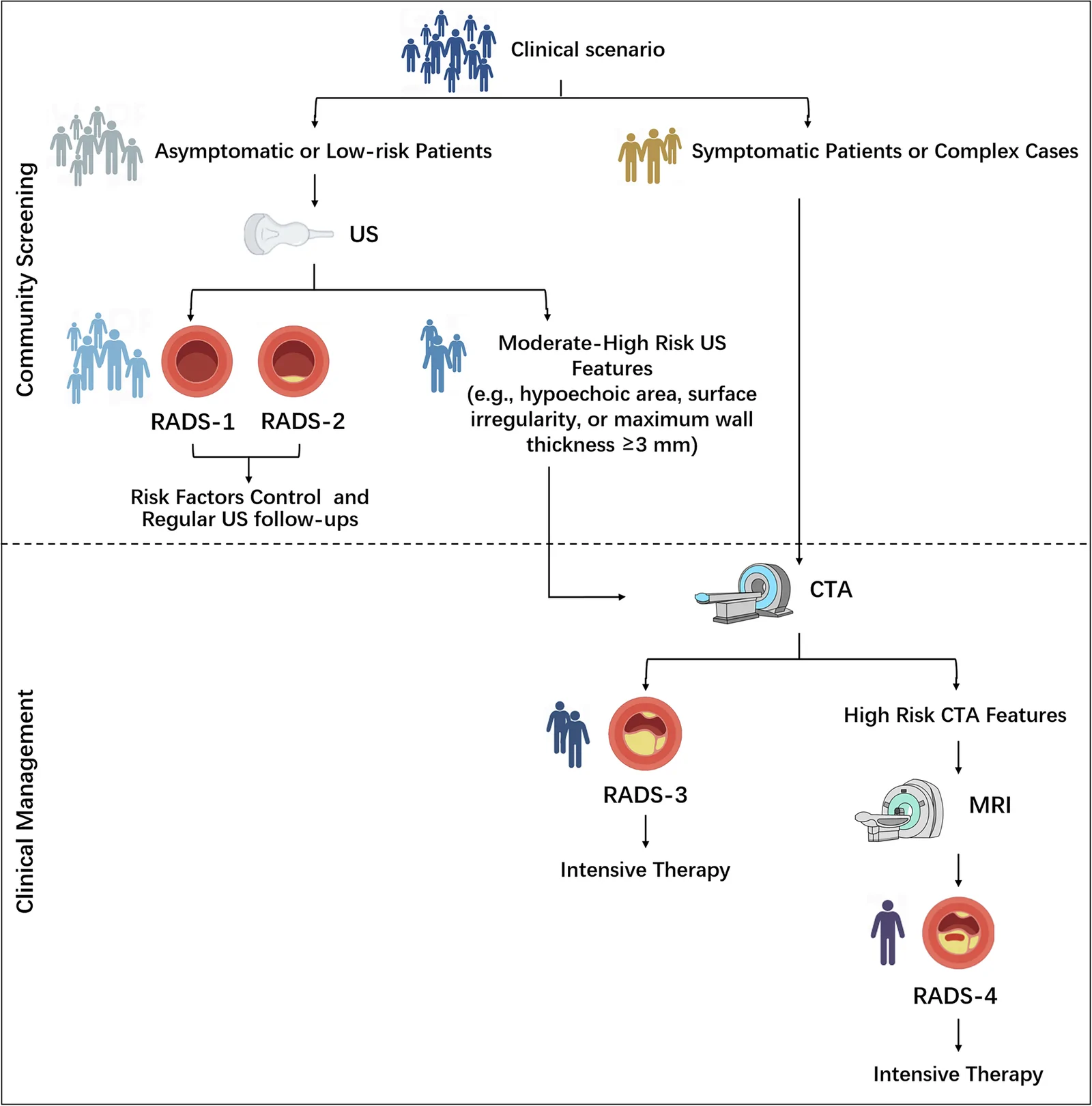
Proposed imaging pathway based on agreement data

Based on our findings, we propose a hypothesis‑generated stratified imaging pathway (Fig. 5). For patients with suspected carotid artery disease in whom initial US identifies low‑risk plaques, US provides sufficient information for risk stratification at minimal cost. Its reliability in identifying low‑risk plaques supports its role in this patient population. Nevertheless, our data show that US has only fair agreement with MRI for detecting IPH (κ, 0.397). Since IPH is a critical feature defining RADS‑4 plaques and directly influences decisions for intensive medical therapy or revascularization, US cannot be relied upon to exclude high‑risk lesions. Accordingly, the proposed pathway (Fig. 5) uses US solely to identify plaques that are unequivocally low‑risk (RADS 1‑2). When US reveals any feature suggestive of moderate or high risk, such as hypoechoic areas (possibly indicating lipid core or IPH), surface irregularity, or maximum wall thickness ≥ 3 mm, the pathway explicitly recommends confirmatory imaging with CTA or MRI. This design respects the known limitations of US while preserving its valuable role as a cost‑effective screening tool for low‑risk populations. For symptomatic patients or those with moderate‑risk US findings, a stepwise approach is warranted. CTA offers an excellent balance of comprehensive anatomical information and reliability for treatment planning. Its superior performance in ulcer detection and stenosis quantification, combined with better accessibility than MRI, makes it well‑suited for this patient cohort. For complex cases, equivocal findings, or when high‑risk features are suspected, MRI should be employed as the definitive modality. Its unparalleled soft‑tissue characterization resolves diagnostic uncertainties and provides the most comprehensive vulnerability assessment, particularly valuable when revascularization is being considered [19, 20]. This hypothesized pathway attempts to balance the strengths and limitations of each modality based on agreement data. However, we emphasize that no formal cost‑effectiveness analysis has been performed, and the proposed strategy should be tested in future prospective studies (Fig. 5).

Our study has limitations. First, this study was a single-center retrospective study, which potentially limited the generalizability of the results. In the future, we will conduct multi-center studies to validate our results. Second, the use of MRI expert consensus as the reference standard warrants critical consideration. While high-resolution MRI is widely accepted as the best non‑invasive modality for carotid plaque characterization, it is not a true histological gold standard. In this study, the same imaging modality (MRI) served both as the test and the reference for agreement calculations, which may introduce a systematic bias that favors MRI over US and CTA. Consequently, the superior agreement observed for MRI should be interpreted as reflecting the higher internal consistency of MRI interpretation rather than necessarily proving its absolute diagnostic superiority. Our conclusions regarding modality performance are therefore strictly grounded in the agreement and reclassification improvement data presented, and we avoid claiming that MRI is definitively superior in all clinical contexts. Future studies incorporating histopathological validation are needed to confirm these findings and to determine the true diagnostic accuracy of each modality. Third, the study population was selected from patients who underwent all three imaging examinations within one month, which likely enriched the cohort for higher‑risk or more complex disease. This is evident from the high prevalence of prior stroke (78.0%), hypertension (60.8%), and smoking history (76.8%) in our sample, as well as the predominance of RADS 3–4 plaques (70.6%). Consequently, the excellent agreement observed for low‑risk plaques (RADS 1–2) was derived from a small subset within an enriched high‑risk cohort and may not be generalizable to a true community‑based screening population with lower disease prevalence. Therefore, our recommendation that US is reliable for identifying low‑risk plaques should be interpreted within the context of secondary prevention or diagnostic workup in patients with suspected carotid disease, and not as an endorsement for mass screening in asymptomatic low‑risk individuals. Future prospective studies in general or primary care populations are needed to validate the performance of US in a truly screening setting. Last, the inter‑modality comparisons reported in Tables 1 and 2 (upper half) were performed using different readers for each modality. Therefore, the observed agreement reflects both modality‑specific technical differences and reader‑dependent variability. A pure measure of inter‑modality agreement would ideally require the same reader to interpret all three imaging techniques; however, this was not feasible in our study because a single radiologist rarely masters all three modalities at an expert level for detailed plaque characterization. Readers should interpret the upper half of the tables as a combined effect. Future work using the same readers for all modalities, perhaps with dedicated training across techniques, would help separate modality‑specific from reader‑dependent variability.

## Conclusion

The Carotid Plaque-RADS demonstrated favorable reliability for lower-risk plaques across US, CTA, and MRI, and across reader experience levels, supporting the use of US as a first-line assessment tool in patients with suspected carotid artery disease. For higher-risk categories, MRI was the preferred modality for comprehensive plaque characterization, while CTA provided valuable balanced information for treatment planning. Reader experience significantly impacts consistency, especially in US, but structured training with defined case volumes can effectively reduce this variability. These findings may help inform imaging selection for carotid artery disease, pending prospective validation and cost‑effectiveness analysis.

## Supplementary information

## Data Availability

The data that support the findings of this study are available from the corresponding author upon reasonable request.

## Abbreviations

CI: Confidence interval
CTA: Computed tomography angiography
ICC: Intraclass correlation coefficient
IPH: Intraplaque hemorrhage
MRI: Magnetic resonance imaging
MWT: Maximum wall thickness
Plaque-RADS: Carotid plaque reporting and data system
US: Ultrasound

## References

[1] Bos D, Arshi B, van den Bouwhuijsen QJA et al (2021) Atherosclerotic carotid plaque composition and incident stroke and coronary events. J Am Coll Cardiol 77:1426–1435. 10.1016/j.jacc.2021.01.038 33736825

[2] Vergallo R, Park SJ, Stone GW et al (2025) Vulnerable or high-risk plaque: a JACC: cardiovascular imaging position statement. JACC Cardiovasc Imaging 18:709–740. 10.1016/j.jcmg.2024.12.004 40019413

[3] Saba L, Cau R, Murgia A et al (2024) Carotid plaque-RADS: a novel stroke risk classification system. JACC Cardiovasc Imaging 17:62–75. 10.1016/j.jcmg.2023.09.005 37823860

[4] Huang Z, Cheng XQ, Lu RR et al (2025) Incremental prognostic value of carotid plaque-RADS over stenosis degree in relation to stroke risk. JACC Cardiovasc Imaging 18:77–89. 10.1016/j.jcmg.2024.07.004 39243231

[5] Johri AM, Nambi V, Naqvi TZ et al (2020) Recommendations for the assessment of carotid arterial plaque by ultrasound for the characterization of atherosclerosis and evaluation of cardiovascular risk: from the American Society of Echocardiography. J Am Soc Echocardiogr 33:917–933. 10.1016/j.echo.2020.04.021 32600741

[6] Saba L, Yuan C, Hatsukami TS et al (2018) Carotid artery wall imaging: perspective and guidelines from the ASNR vessel wall imaging study group and expert consensus recommendations of the American Society of Neuroradiology. AJNR Am J Neuroradiol 39:E9–E31. 10.3174/ajnr.A5488 29326139 PMC7410574

[7] Naqvi TZ (2024) Multimodality imaging classification for carotid plaque assessment. JACC Cardiovasc Imaging 17:76–78. 10.1016/j.jcmg.2023.11.004 38176850

[8] Saba L, Scicolone R, Chabert GL et al (2025) Carotid plaque-RADS: inter-and intra-reader agreement and learning curve analysis in computed tomography angiography. AJNR Am J Neuroradiol. 10.3174/ajnr.A8769 PMC12633665 40194857

[9] Cassola N, Baptista-Silva JC, Nakano LC et al (2022) Duplex ultrasound for diagnosing symptomatic carotid stenosis in the extracranial segments. Cochrane Database Syst Rev 7:CD013172. 10.1002/14651858.CD013172.pub2 35815652 PMC9272405

[10] Golemati S, Cokkinos DD (2022) Recent advances in vascular ultrasound imaging technology and their clinical implications. Ultrasonics 119:106599. 10.1016/j.ultras.2021.106599 34624584

[11] Baradaran H, Al-Dasuqi K, Knight-Greenfield A et al (2017) Association between carotid plaque features on CTA and cerebrovascular ischemia: a systematic review and meta-analysis. AJNR Am J Neuroradiol 38:2321–2326. 10.3174/ajnr.A5436 29074638 PMC7963758

[12] Dakis K, Nana P, Athanasios C et al (2023) Carotid plaque vulnerability diagnosis by CTA versus MRA: a systematic review. Diagnostics (Basel). 10.3390/diagnostics13040646 PMC9955971 36832133

[13] Baradaran H, Gupta A (2020) Carotid vessel wall imaging on CTA. AJNR Am J Neuroradiol 41:380–386. 10.3174/ajnr.A6403 32029468 PMC7077912

[14] Brinjikji W, Huston J 3rd, Rabinstein AA et al (2016) Contemporary carotid imaging: from degree of stenosis to plaque vulnerability. J Neurosurg 124:27–42. 10.3171/2015.1.JNS142452 26230478

[15] Pakizer D, Kozel J, Taffe P et al (2024) Diagnostic accuracy of carotid plaque instability by noninvasive imaging: a systematic review and meta-analysis. Eur Heart J Cardiovasc Imaging 25:1325–1335. 10.1093/ehjci/jeae144 38953552

[16] van Dam-Nolen DHK, Truijman MTB, van der Kolk AG et al (2022) Carotid plaque characteristics predict recurrent ischemic stroke and TIA: the PARISK (plaque at RISK) study. JACC Cardiovasc Imaging 15:1715–1726. 10.1016/j.jcmg.2022.04.003 36202450

[17] Zuo L, Kavousi M, Mo H et al (2025) Evolution of subclinical carotid atherosclerotic plaque composition using serial MRI in the rotterdam study. Radiology 315:e242248. 10.1148/radiol.242248 40459414

[18] Sun J, Zhao XQ, Balu N et al (2017) Carotid plaque lipid content and fibrous cap status predict systemic CV outcomes: the MRI substudy in AIM-HIGH. JACC Cardiovasc Imaging 10:241–249. 10.1016/j.jcmg.2016.06.017 28279371 PMC5347460

[19] Culleton S, Baradaran H, Kim SE et al (2022) MRI detection of carotid intraplaque hemorrhage and postintervention cognition. AJNR Am J Neuroradiol 43:1762–1769. 10.3174/ajnr.A7701 36357151

[20] McNally JS, McLaughlin MS, Hinckley PJ et al (2015) Intraluminal thrombus, intraplaque hemorrhage, plaque thickness, and current smoking optimally predict carotid stroke. Stroke 46:84–90. 10.1161/STROKEAHA.114.006286 25406146

