# Supplementary material for: Inter-reader agreement in multimodality imaging of carotid plaque-RADS: considerations for modality selection
Source: Insights Imaging. 2026 Sep 2;17:217. doi: 10.1186/s13244-026-02393-3 (PMC13538297; doi:10.1186/s13244-026-02393-3)
Supplement: Supplementary file 1 [file 13244_2026_2393_MOESM1_ESM.pdf]

Inter-reader Agreement in Multimodality Imaging of Carotid Plaque-RADS: Considerations for Modality Selection

ELECTRONIC SUPPLEMENTARY MATERIAL

Insights Imaging (2026) Zhang J, Yan Y, Xing Y, Bai L, Sa R, Wang L

Supplementary File S1 Examination protocols for US, CTA, and MRI

All US examinations were performed using linear-array transducers (frequency range: 3–10 MHz) on commercially available systems, including the IU22 and IE33 (Philips Medical Solutions) and the Mindray Resona 5 (Mindray). With patients in the supine position and neck slightly extended, the carotid arteries were systematically imaged from the proximal common carotid artery to the internal carotid artery, acquiring both longitudinal and transverse plane images. Grayscale, color Doppler, and spectral Doppler evaluations were performed to assess plaque morphology, surface characteristics, vascular patency, and hemodynamic parameters. All examinations included stored cine loops and still images for subsequent offline analysis.

CTA examinations were performed using either a SOMATOM 128-slice scanner (Siemens Healthineers) or a Revolution 256-slice scanner (GE Healthcare). The scanning range extended from the ascending aortic arch to the cranial vertex to ensure complete coverage of the extracranial and intracranial carotid segments. Non-contrast scans were initially acquired to evaluate calcification burden, followed by contrast-enhanced angiography using a bolus-tracking technique with 60–80 mL of iodinated contrast medium (370–400 mgI/mL) injected at 4–5 mL/s. Scan parameters were optimized for vascular imaging: tube voltage 100–120 kV, automatic tube current modulation, and collimation of 128 × 0.6 mm or 256 × 0.625 mm depending on the scanner type. Images were reconstructed with 0.5–0.75 mm slice thickness and 0.4–0.5 mm increments using both standard and bone kernel algorithms.

MRI examinations were performed on 3.0-T systems (Achieva TX [Philips Healthcare] or Skyra [Siemens Healthineers]) using dedicated 8-channel carotid surface coils. The imaging protocol was optimized for plaque characterization and included the following sequences: three-dimensional time-of-flight (3D-TOF); T1- and T2-weighted black-blood sequences for plaque morphology and composition; and contrast-enhanced T1-weighted imaging acquired after intravenous administration of a gadolinium-based contrast agent to assess fibrous cap integrity. The field of

view was centered on the carotid bifurcation or regions of significant stenosis identified from prior US or CTA. High-resolution parameters were used: matrix ≥256×256, slice thickness ≤2.0 mm, yielding an in-plane resolution of ~0.5×0.5 mm^2. Representative sequence parameters were: TOF (TR/TE: 20/3.5 ms), Black-blood T1WI (TR/TE: 800/10 ms).

Supplementary Table S1 Criteria used for assessing plaque features across different imaging modalities

	Ultrasound	CTA	MRI
Presence of plaque	Focal or diffuse intima-media thickness ≥1.5 mm or 50% of the surrounding area[1]	Circumferential or eccentric wall thickening with abnormal attenuation.	Focal or concentric wall thickening with abnormal signal intensity on T1WI.
Maximum wall thickness	The maximum distance from the lumen-intima interface to the media-adventitia interface on a longitudinal view.[2]	The maximum distance from the lumen boundary to the outer wall boundary measured on multi-planar reformatted images orthogonal to the vessel centerline.[3]	The maximum distance from the lumen boundary to the outer wall boundary measured on T1WI.[4]
Ulceration	A distinct recess or crater extending into the plaque from the lumen surface, with a depth > 1 mm.[5]	Contrast outpouching ≥1 mm.[6]	A juxtaluminal focal depression into the plaque.[7]
Fibrous cap integrity	a focal discontinuity of hyperechoic lines on the plaque surface.[8]	Not applicable.	A ruptured cap is defined by a focal interruption in the enhancing plaque surface on post-contrast T1WI.[9]
Intraplaque hemorrhage	A juxtaluminal black area.[10]	An area < 25 HU and/or the presence	A focal, hyperintense signal within

		of positive rim sign[11]	the plaque on T1WI and does not enhance post-contrast.[12]
Intraluminal thrombus	A floating thrombus dynamically changing its position over time[13]	Doughnut sign[14]	A noncontrast filling defect outlined by lumen contrast.[15]

Reference

1. Johri AM, Nambi V, Naqvi TZ, et al. Recommendations for the Assessment of Carotid Arterial Plaque by Ultrasound for the Characterization of Atherosclerosis and Evaluation of Cardiovascular Risk: From the American Society of Echocardiography. *J Am Soc Echocardiogr* 2020; 33: 917-933. 2020/07/01. DOI: 10.1016/j.echo.2020.04.021.
2. Zhao X, Hippe DS, Li R, et al. Prevalence and Characteristics of Carotid Artery High-Risk Atherosclerotic Plaques in Chinese Patients With Cerebrovascular Symptoms: A Chinese Atherosclerosis Risk Evaluation II Study. *J Am Heart Assoc* 2017; 6 2017/09/02. DOI: 10.1161/JAHA.117.005831.
3. Gupta A, Baradaran H, Kamel H, et al. Evaluation of computed tomography angiography plaque thickness measurements in high-grade carotid artery stenosis. *Stroke* 2014; 45: 740-745. 2014/02/06. DOI: 10.1161/STROKEAHA.113.003882.
4. Selwaness M, Bos D, van den Bouwhuijsen Q, et al. Carotid Atherosclerotic Plaque Characteristics on Magnetic Resonance Imaging Relate With History of Stroke and Coronary Heart Disease. *Stroke* 2016; 47: 1542-1547. 2016/05/12. DOI: 10.1161/STROKEAHA.116.012923.
5. ten Kate GL, van Dijk AC, van den Oord SC, et al. Usefulness of contrast-enhanced ultrasound for detection of carotid plaque ulceration in patients with symptomatic carotid atherosclerosis. *Am J Cardiol* 2013; 112: 292-298. 2013/04/17. DOI: 10.1016/j.amjcard.2013.03.028.
6. Anderson GB, Ashforth R, Steinke DE, et al. CT angiography for the detection and characterization of carotid artery bifurcation disease. *Stroke* 2000; 31: 2168-2174. 2000/09/08. DOI: 10.1161/01.str.31.9.2168.
7. den Hartog AG, Bovens SM, Koning W, et al. Current status of clinical magnetic resonance imaging for plaque characterisation in patients with carotid artery stenosis. *Eur J Vasc Endovasc Surg* 2013; 45: 7-21. 2012/12/04. DOI: 10.1016/j.ejvs.2012.10.022.
8. Johri AM, Herr JE, Li TY, et al. Novel Ultrasound Methods to Investigate Carotid Artery Plaque Vulnerability. *J Am Soc Echocardiogr* 2017; 30: 139-148. 2016/12/18. DOI: 10.1016/j.echo.2016.11.003.
9. Sun J, Zhao XQ, Balu N, et al. Carotid Plaque Lipid Content and Fibrous Cap Status Predict Systemic CV Outcomes: The MRI Substudy in AIM-HIGH. *JACC Cardiovasc Imaging* 2017; 10: 241-249. 2017/03/11. DOI: 10.1016/j.jcmg.2016.06.017.
10. van Dam-Nolen DHK, Truijman MTB, van der Kolk AG, et al. Carotid Plaque Characteristics Predict Recurrent Ischemic Stroke and TIA: The PARISK (Plaque At RISK) Study. *JACC Cardiovasc Imaging* 2022; 15: 1715-1726. 2022/10/07. DOI: 10.1016/j.jcmg.2022.04.003.
11. Saba L, Scicolone R, Chabert GL, et al. Carotid Plaque-RADS: Inter- and Intrareader Agreement and Learning Curve Analysis in CTA. *AJNR Am J Neuroradiol* 2025; 46: 2009-2017. 2025/04/08. DOI: 10.3174/ajnr.A8769.

12. van der Toorn JE, Bos D, Ikram MK, et al. Carotid Plaque Composition and Prediction of Incident Atherosclerotic Cardiovascular Disease. *Circ Cardiovasc Imaging* 2022; 15: e013602. 2022/02/25. DOI: 10.1161/CIRCIMAGING.121.013602.
13. Morelli N, Rota E, Spallazzi M, et al. Ultrasound in free-floating thrombus of the carotid artery: the best diagnostic tool to detect this under estimated condition? *Acta Neurol Belg* 2014; 114: 65-66. 2012/11/01. DOI: 10.1007/s13760-012-0149-3.
14. Alhowaish TS, Alhamadh MS, Alsulayhim A, et al. Intraluminal Thrombus of the Extracranial Cerebral Arteries in Acute Ischemic Stroke: Manifestations, Treatment Strategies, and Outcome. *Vasc Health Risk Manag* 2024; 20: 1-12. 2024/01/09. DOI: 10.2147/VHRM.S435227.
15. McNally JS, McLaughlin MS, Hinckley PJ, et al. Intraluminal thrombus, intraplaque hemorrhage, plaque thickness, and current smoking optimally predict carotid stroke. *Stroke* 2015; 46: 84-90. 2014/11/20. DOI: 10.1161/STROKEAHA.114.006286.

Supplemental Table S2 The distribution of Plaque-RADS categories across the five subsets

	Subset 1 (n = 193)	Subset 2 (n = 195)	Subset 3 (n = 199)	Subset 4 (n = 198)	Subset 5 (n = 202)	*P* value
Plaque-RADS						0.981
RADS-1	4 (2.1)	5 (2.6)	5 (2.5)	5 (2.5)	6 (3.0)	
RADS-2	45 (23.3)	55 (28.2)	50 (25.1)	59 (29.8)	56 (27.7)	
RADS-3	79 (40.9)	72 (36.9)	73 (36.7)	75 (37.9)	77 (38.1)	
RADS-4	65 (33.7)	63 (32.3)	71 (35.7)	59 (29.8)	63 (31.2)	

Plaque-RADS, Carotid Plaque Reporting and Data System.

Supplemental Table S3 Inter-reader agreement of the major features of Plaque-RADS (one plaque per artery)

	Presence of plaque	MWT	Ulceration	Fibrous cap integrity	IPH	Intraluminal thrombus
Based on different imaging modalities						
Be						
US & CTA	0.786 (0.742, 0.850)	0.751 (0.719, 0.778)	0.164 (0.088, 0.249)	NA	0.346 (0.268, 0.421)	0.575 (0.359, 0.786)
CTA & MRI	0.780 (0.716, 0.838)	0.714 (0.687, 0.746)	0.485 (0.400, 0.552)	NA	0.195 (0.131, 0.257)	0.575 (0.359, 0.786)
US & MRI	0.763 (0.703, 0.858)	0.657 (0.615, 0.690)	0.177 (0.104, 0.271)	0.286 (0.201, 0.351)	0.236 (0.176, 0.309)	0.339 (0.116, 0.467)
In						
US & CTA	0.852 (0.805, 0.914)	0.756 (0.724, 0.789)	0.259 (0.159, 0.337)	NA	0.511 (0.439, 0.592)	0.490 (0.305, 0.646)
CTA & MRI	0.838 (0.794, 0.904)	0.776 (0.730, 0.821)	0.682 (0.614, 0.753)	NA	0.446 (0.370, 0.519)	0.595 (0.421, 0.733)
US & MRI	0.828 (0.767, 0.904)	0.713 (0.678, 0.751)	0.312 (0.192, 0.400)	0.362 (0.269, 0.432)	0.206 (0.145, 0.272)	0.515 (0.291. 0.667)
Ex						
US & CTA	0.929 (0.891, 0.982)	0.746 (0.718, 0.778)	0.414 (0.309, 0.524)	NA	0.436 (0.342, 0.504)	0.573 (0.337, 0.723)
CTA & MRI	0.922 (0.887, 0.979)	0.756 (0.732, 0.779)	0.880 (0.843, 0.925)	NA	0.571 (0.519, 0.620)	0.754 (0.576, 0.876)
US & MRI	0.887 (0.838, 0.940)	0.712 (0.674, 0.743)	0.426 (0.318, 0.523)	0.464 (0.366, 0.549)	0.295 (0.209, 0.364)	0.574 (0.359. 0.785)
Based on different reader experiences						
US						
Be & In	0.957 (0.927, 0.993)	0.824 (0.803, 0.844)	0.585 (0.378, 0.772)	0.409 (0.297, 0.512)	0.379 (0.312, 0.446)	0.654 (0.477, 0.777)
In & Ex	0.954 (0.924, 0.999)	0.831 (0.797, 0.855)	0.655 (0.466, 0.780)	0.650 (0.561, 0.740)	0.511 (0.439, 0.592)	0.721 (0.596, 0.842)
Be & Ex	0.920 (0.872, 0.967)	0.745 (0.709, 0.770)	0.563 (0.396, 0.722)	0.543 (0.452, 0.634)	0.319 (0.242, 0.395)	0.518 (0.294, 0.670)
CTA						
Be & In	0.963 (0.931, 0.984)	0.799 (0.758, 0.830)	0.743 (0.678, 0.792)	NA	0.571 (0.519, 0.620)	0.654 (0.477, 0.777)

In & Ex	0.963 (0.928, 0.992)	0.839 (0.819, 0.863)	0.894 (0.849, 0.939)	NA	0.536 (0.442, 0.604)	0.721 (0.596, 0.842)
Be & Ex	0.915 (0.880, 0.958)	0.756 (0.732, 0.778)	0.660 (0.585, 0.725)	NA	0.511 (0.439, 0.592)	0.600 (0.356, 0.766)
MRI						
Be & In	0.960 (0.921, 0.992)	0.873 (0.848, 0.896)	0.691 (0.626, 0.770)	0.762 (0.703, 0.808)	0.889 (0.845, 0.936)	0.721 (0.596, 0.842)
In & Ex	0.959 (0.904, 0.990)	0.888 (0.872, 0.903)	0.864 (0.802, 0.904)	0.849 (0.812, 0.910)	0.895 (0.850, 0.942)	0.854 (0.706, 0.977)
Be & Ex	0.904 (0.846, 0.959)	0.789 (0.765, 0.813)	0.635 (0.558, 0.722)	0.619 (0.540, 0.686)	0.781 (0.721, 0.832)	0.695 (0.521, 0.833)

Data are kappa scores or intraclass correlation coefficient. Data in parentheses are 95% confidence intervals. MWT, maximal wall thickness; IPH, intra-plaque hemorrhage; Be, beginner readers; In, intermediate readers; Ex, expert readers; US, ultrasound, CTA, computed tomography angiography, MRI, magnetic resonance imaging; NA, not applicable.

Given that CTA could not directly evaluate fibrous cap integrity, this parameter was not assessed in the CTA analysis.

Supplemental Table S4 Inter-reader agreement for assigning Plaque-RADS categories (one plaque per artery)

	RADS	RADS-1	RADS-2	RADS-3	RADS-4
Based on different imaging modalities					
Be					
US & CTA	0.566 (0.532, 0.607)	0.870 (0.822, 0.929)	0.748 (0.705, 0.789)	0.493 (0.437, 0.549)	0.259 (0.202, 0.329)
CTA & MRI	0.600 (0.572, 0.635)	0.915 (0.866, 0.963)	0.754 (0.713, 0.801)	0.489 (0.445, 0.537)	0.478 (0.417, 0.541)
US & MRI	0.441 (0.386, 0.480)	0.824 (0.775, 0.887)	0.683 (0.633, 0.722)	0.316 (0.243, 0.366)	0.185 (0.123, 0.252)
In					
US & CTA	0.634 (0.600, 0.673)	0.888 (0.850, 0.944)	0.819 (0.787, 0.857)	0.559 (0.513, 0.613)	0.411 (0.352, 0.483)
CTA & MRI	0.648 (0.612, 0.684)	0.810 (0.759, 0.900)	0.791 (0.736, 0.833)	0.557 (0.503, 0.616)	0.569 (0.515, 0.619)
US & MRI	0.526 (0.486, 0.566)	0.773 (0.714, 0.849)	0.746 (0.704, 0.784)	0.418 (0.369, 0.468)	0.344 (0.288, 0.413)
Ex					
US & CTA	0.682 (0.655, 0.723)	0.833 (0.774, 0.900)	0.849 (0.816, 0.880)	0.605 (0.567, 0.654)	0.529 (0.467, 0.577)
CTA & MRI	0.800 (0.766, 0.831)	0.887 (0.838, 0.959)	0.881 (0.851, 0.915)	0.744 (0.703, 0.786)	0.762 (0.713, 0.798)
US & MRI	0.597 (0.554, 0.632)	0.833 (0.774, 0.900)	0.825 (0.780, 0.865)	0.491 (0.442, 0.537)	0.435 (0.365, 0.491)
Based on different reader experiences					
US					
Be & In	0.681 (0.640, 0.709)	0.858 (0.816, 0.919)	0.769 (0.728, 0.804)	0.644 (0.578, 0.691)	0.488 (0.403, 0.572)

In & Ex	0.810 (0.777, 0.838)	0.984 (0.966, 0.999)	0.924 (0.903, 0.945)	0.768 (0.725, 0.810)	0.628 (0.563, 0.685)
Be & Ex	0.641 (0.588, 0.677)	0.843 (0.799, 0.908)	0.736 (0.689, 0.776)	0.592 (0.536, 0.647)	0.451 (0.377, 0.518)
CTA					
Be & In	0.698 (0.665, 0.731)	0.933 (0.891, 0.968)	0.840 (0.803, 0.872)	0.638 (0.589, 0.674)	0.535 (0.479, 0.588)
In & Ex	0.847 (0.820, 0.872)	0.903 (0.854, 0.949)	0.900 (0.876, 0.936)	0.813 (0.778, 0.845)	0.812 (0.774, 0.851)
Be & Ex	0.678 (0.647, 0.717)	0.945 (0.901, 0.982)	0.809 (0.777, 0.843)	0.601 (0.561, 0.648)	0.550 (0.496, 0.616)
MRI					
Be & In	0.721 (0.685, 0.755)	0.858 (0.807, 0.922)	0.876 (0.842, 0.903)	0.625 (0.580, 0.668)	0.644 (0.592, 0.703)
In & Ex	0.879 (0.858, 0.903)	0.940 (0.904, 0.979)	0.959 (0.937, 0.975)	0.836 (0.802, 0.870)	0.841 (0.806, 0.872)
Be & Ex	0.715 (0.679, 0.748)	0.870 (0.823, 0.940)	0.882 (0.839, 0.911)	0.616 (0.570, 0.664)	0.629 (0.589, 0.667)

Data are kappa scores or intraclass correlation coefficient. Data in parentheses are 95% confidence intervals. Be, beginner readers; In, intermediate readers; Ex, expert readers; US, ultrasound, CTA, computed tomography angiography, MRI, magnetic resonance imaging.

Supplemental Table S5 Inter-reader agreement across sequential subsets (one plaque per artery)

κ statistics	Subset 1	Subset 2	Subset 3	Subset 4	Subset 5
US beginner reader	0.287 (0.204, 0.391)	0.367 (0.285, 0.463)	0.443 (0.361, 0.538)	0.496 (0.417, 0.588)	0.503 (0.419, 0.587)
US intermediate reader	0.436 (0.341, 0.515)	0.503 (0.425, 0.595)	0.569 (0.469, 0.662)	0.562 (0.479, 0.643)	0.572 (0.493, 0.654)
US expert reader	0.483 (0.399, 0.575)	0.557 (0.467, 0.646)	0.619 (0.521, 0.700)	0.605 (0.526, 0.690)	0.618 (0.536, 0.698)
CTA beginner reader	0.487 (0.402, 0.568)	0.579 (0.490, 0.668)	0.642 (0.545, 0.718)	0.634 (0.559, 0.714)	0.645 (0.575, 0.738)
CTA intermediate reader	0.594 (0.517, 0.672)	0.703 (0.623, 0.800)	0.722 (0.643, 0.789)	0.728 (0.663, 0.800)	0.734 (0.671, 0.800)
CTA expert reader	0.693 (0.616, 0.767)	0.787 (0.716, 0.853)	0.809 (0.734, 0.867)	0.820 (0.753, 0.887)	0.819 (0.764, 0.881)
MRI beginner reader	0.586 (0.483, 0.692)	0.675 (0.597, 0.733)	0.721 (0.656, 0.792)	0.734 (0.662, 0.812)	0.750 (0.667, 0.832)
MRI intermediate reader	0.793 (0.731, 0.867)	0.866 (0.810, 0.925)	0.884 (0.830, 0.933)	0.896 (0.840, 0.956)	0.897 (0.849, 0.956)

Data are kappa scores or intraclass correlation coefficient. Data in parentheses are 95% confidence intervals. US, ultrasound, CTA, computed tomography angiography, MRI, magnetic resonance imaging.

Supplemental Table S6 Inter-reader agreement across sequential subsets (modality-specific expert references)

κ statistics	Subset 1	Subset 2	Subset 3	Subset 4	Subset 5
US expert reference					
US beginner reader	0.465 (0.363, 0.567)	0.456 (0.352, 0.560)	0.669 (0.581, 0.757)	0.753 (0.673, 0.833)	0.765 (0.687, 0.843)
US intermediate reader	0.637 (0.545, 0.729)	0.697 (0.605, 0.789)	0.953 (0.916, 0.990)	0.949 (0.910, 0.988)	0.952 (0.989, 0.915)
CTA expert reference					
CTA beginner reader	0.592 (0.500, 0.684)	0.597 (0.507, 0.687)	0.709 (0.627, 0.791)	0.747 (0.669, 0.825)	0.765 (0.689, 0.841)
CTA intermediate reader	0.722 (0.642, 0.802)	0.812 (0.743, 0.881)	0.896 (0.843, 0.949)	0.908 (0.857, 0.959)	0.887 (0.832, 0.942)
MRI expert reference					
MRI beginner reader	0.582 (0.486, 0.678)	0.662 (0.574, 0.750)	0.726 (0.646, 0.806)	0.741 (0.663, 0.819)	0.752 (0.676, 0.828)
MRI intermediate reader	0.798 (0.725, 0.871)	0.864 (0.803, 0.925)	0.889 (0.834, 0.944)	0.896 (0.843, 0.949)	0.898 (0.847,0.949)

Data are kappa scores or intraclass correlation coefficient. Data in parentheses are 95% confidence intervals. US, ultrasound, CTA, computed tomography angiography, MRI, magnetic resonance imaging.

Supplemental Table 7 Inter-reader agreement for Plaque-RADS 3 and 4 subcategories

	RADS 3a	RADS 3b	RADS 3c	RADS 4a	RADS 4b	RADS 4c
Based on different imaging modalities						
Be						
US & CTA	NA	NA	0.145 (0.063, 0.227)	0.350 (0.283, 0.417)	NA	0.505 (0.340, 0.670)
CTA & MRI	NA	NA	0.498 (0.412, 0.584)	0.398 (0.337, 0.459)	NA	0.612 (0.437, 0.786)
US & MRI	NA	NA	0.161 (0.071, 0.251)	0.239 (0.168, 0.310)	0.279 (0.199, 0.359)	0.416 (0.244, 0.588)
In						
US & CTA	NA	NA	0.269 (0.167, 0.371)	0.345 (0.267, 0.423)	NA	0.585 (0.430, 0.740)
CTA & MRI	NA	NA	0.652 (0.572, 0.732)	0.515 (0.442, 0.588)	NA	0.675 (0.516, 0.834)
US & MRI	NA	NA	0.297 (0.191, 0.403)	0.295 (0.222, 0.368)	0.361 (0.273, 0.449)	0.522 (0.361. 0.683)
Ex						
US & CTA	NA	NA	0.399 (0.293, 0.505)	0.426 (0.348, 0.504)	NA	0.602 (0.412, 0.765)
CTA & MRI	NA	NA	0.843 (0.786, 0.900)	0.674 (0.611, 0.737)	NA	0.787 (0.669, 0.905)
US & MRI	NA	NA	0.414 (0.306, 0.522)	0.285 (0.207, 0.363)	0.469 (0.382, 0.555)	0.596 (0.441. 0.751)
Based on different reader experiences						
US						
Be & In	NA	NA	0.548 (0.368, 0.728)	0.384 (0.313, 0.455)	0.412 (0.304, 0.520)	0.644 (0.491, 0.797)
In & Ex	NA	NA	0.618 (0.459, 0.777)	0.510 (0.437, 0.583)	0.558 (0.476, 0.640)	0.733 (0.587, 0.878)
Be & Ex	NA	NA	0.566 (0.403, 0.729)	0.320 (0.249, 0.391)	0.449 (0.367, 0.531)	0.511 (0.333, 0.689)

CTA						
Be & In	NA	NA	0.703 (0.630, 0.776)	0.576 (0.515, 0.637)	NA	0.705 (0.509, 0.901)
In & Ex	NA	NA	0.857 (0.802, 0.912)	0.634 (0.558, 0.710)	NA	0.793 (0.617, 0.969)
Be & Ex	NA	NA	0.640 (0.562, 0.718)	0.398 (0.337, 0.459)	NA	0.620 (0.438, 0.802)
MRI						
Be & In	0.795 (0.744, 0.846)	0.641 (0.578, 0.704)	0.655 (0.570, 0.730)	0.879 (0.832, 0.926)	0.759 (0.702, 0.816)	0.720 (0.567, 0.872)
In & Ex	0.865 (0.824, 0.906)	0.791 (0.742, 0.840)	0.817 (0.756, 0.878)	0.888 (0.843, 0.933)	0.853 (0.804, 0.902)	0.829 (0.731, 0.927)
Be & Ex	0.763 (0.708, 0.818)	0.651 (0.588, 0.714)	0.599 (0.515, 0.683)	0.774 (0.711, 0.837)	0.614 (0.543, 0.685)	0.658 (0.505, 0.811)

Data are kappa scores or intraclass correlation coefficient. Data in parentheses are 95% confidence intervals. Be, beginner readers; In, intermediate readers; Ex, expert readers; US, ultrasound, CTA, computed tomography angiography, MRI, magnetic resonance imaging; NA, not applicable.

.
